# LGBTQIA+ STEM Day 2025: an interview with Michel Geovanni (Geo) Santiago-Martinez

**DOI:** 10.1038/s42003-025-09185-3

**Published:** 2025-11-18

## Abstract

In this LGBTQIA+ STEM Day, we spoke to Geo Santiago-Martinez (he/him), an Assistant Professor at the University of Connecticut working on Microbial Ecophysiology, and a member of the Advancing Queer and Transgender Equity in Science (AQTES) consortium.

**Figure:**
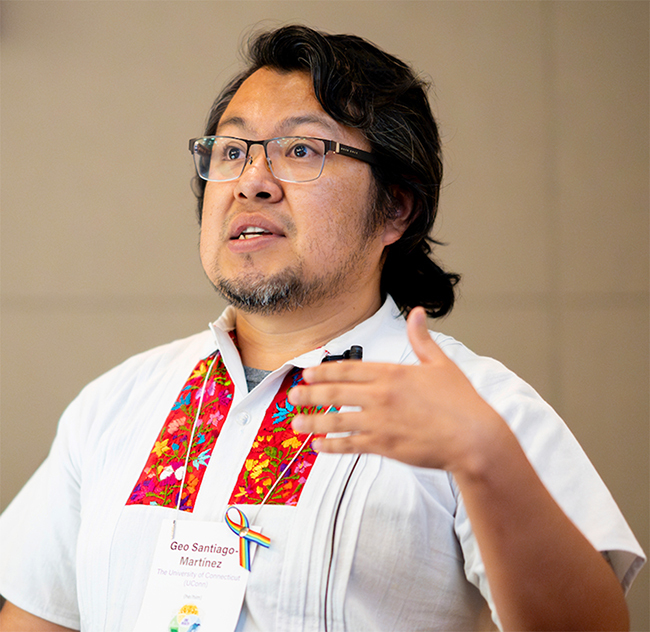
Michel Geovanni (Geo) Santiago-Martinez

Being queer in STEM is an opportunity to see that science can be understood from different perspectives

Please tell me a bit about your research interests.

My research group studies methane-producing microorganisms, or methanogens. We want to understand how these microorganisms produce energy and methane, and how they survive different types of stresses. We work with methanogens from the marine sediment and from the human gut to understand how these organisms are important for the health of ecosystems and humans.

What development in your field are you most excited about?

One of the most exciting developments right now is that methanogens are found inside humans. This finding has sparked a controversial debate on whether these microorganisms are good or bad for human health. Many publications have come out in recent years about human gut methanogens and their potential role. While my background is on environmental methanogens, this development has inspired us to use our expertise to study methanogens associated with human microbiomes.

What are the main barriers for LGBTQ+ researchers right now?

The main barrier right now for LGBTQ+ researchers in the USA is that recently there have been many changes to legislation related to human rights, and LGBTQ+ researchers are losing these rights. This is changing the whole country, but in the case of science, it is affecting early career researchers the most, as there are new barriers that will affect them. This is not specific for this administration, as we have seen these changes to legislation over the years, but these barriers have become more widespread in the last year.

In the case of Mexico, or Latin America, people that are part of the LGBTQ+ community are usually underrepresented and have less access to resources, jobs, and higher education opportunities. Some countries like Mexico are changing, and improving access and providing more opportunities, but it will take some time until we see the effects of these developments.

As a first-generation immigrant from Mexico, and in the current climate, what do you think are the main challenges that queer, Latin American immigrant scientists face right now in the US?

As a person who is an immigrant but also Mexican, indigenous, Latino and LGBTQ+, I can say firsthand that it is difficult to find the right support. In this administration, there have been many changes to immigration, and procedures to obtain visas and legal immigration status are becoming increasingly difficult, and this is something that is affecting researchers at many levels. In my case, I have to do a lot of additional paperwork and spend my own money to keep my immigration status in addition to fulfilling my job responsibilities as a researcher and lecturer. It is changing the face of research in this country, as international students have less access to research opportunities due to the additional immigration barriers.

What has your experience been as a queer researcher in STEM?

In general, I have had positive experiences in labs as a queer researcher, and in my current institution, I have the support I need to be myself and be a good scientist. For me, this is super important because this is the best way to focus on my research and job responsibilities. Having the support to help me feel safe and happy in my workplace is something that is unique and I’m very thankful for that. Because I have that support for myself, I can extend that support and help other people too. The most important thing is getting the support that you need to be yourself in your workplace and then use that support to help others too. When I first started my career, I did not have a role model as it was rare to see someone like me in my position (as a university professor or scientist). Now there is more visibility for queer researchers in STEM. Fortunately, this is something that is changing, although slowly, but now younger scientists have more queer role models.

What can publishers and research institutions do to support LGBTQ+ researchers?

When it comes to publishers, I think having the intention to do something and improve representation is the first step. The second step is inviting more LGBTQ+ researchers to review papers and submit manuscripts. I think this would be very beneficial for the queer community, as sometimes there are a lot of barriers to publish and having access to necessary resources, so inviting more queer people to participate in the peer review process is something publishers can do to support us.

In terms of academia, the first step is for group leaders to promote a safe and inclusive lab environment. I think institutions should offer training, such as workshops, to encourage and train group leaders on how to achieve this. It is important for group leaders to recognise that we should be including and hiring people with diverse backgrounds and perspectives in our labs. Group leaders should also ask their group members what their needs are and adapt their teaching and mentoring styles accordingly.

Moreover, I think institutions can give additional support for researchers with intersectional identities. It is also important for institutions to offer support for researchers struggling with mental health or experiencing challenges in their careers. I think that these support networks should exist in all academic institutions. In my current institution (the University of Connecticut), we have internal committees to support students within our department, but also external mentoring committees. This is important because if you are in the same department, sometimes it’s difficult to talk about some sensitive topics. I usually encourage my students to look for external mentors so they can learn from people with different backgrounds and experiences. I think this is the way that we can learn more about how we can do science in a better and inclusive place.

Please tell me more about the consortium Advancing Queer and Transgender Equity in Science (AQTES).

This is a consortium of people who identify as part of the LGBTQ+ community, and we want to provide help to promote more inclusive environments for queer people in STEM. We published a paper with guidelines to improve representation when hiring people. For queer, and specifically trans people, the experience of applying for a faculty job can sometimes be exclusionary. We offer resources or tools for people to understand how difficult it can be to undergo a hiring process. For example, having a different legal name can be a barrier for trans people looking for a job. I invite everyone to encourage institutions to follow these guidelines in the hiring process.

Are there any other initiatives to promote intersectionality in STEM that you are excited about?

Yes, I am excited about the Archaea Power Hour community. This is a consortium where we aim to connect archaea researchers around the world while promoting an inclusive environment. We have a seminar series and networking sessions to meet researchers worldwide, so people can learn about archaea research, job opportunities, start collaborations, and share resources. We offer participants that don’t feel comfortable speaking in English the option to present their research in other languages, and we support them with translation efforts to achieve a more inclusive environment for non-native English speakers. We are currently trying to obtain financial support to broaden the impact of this initiative. We also have a subcommittee to promote diversity, equity and inclusion, and some of the organisers are part of the LGBTQ+ community. We use this initiative to share our science, but also to learn from people with diverse experiences and thus promote safe and inclusive environments for all researchers. We also collaborate with ArchaeaBio to promote opportunities and resources worldwide.

What is your advice for queer students who want to pursue a career in science?

My main advice is: “find your support community”, which might come in different shapes. While your institution might not have specific support for queer people, you may find support in other communities. In my case, I have found support attending or participating in initiatives with Spanish-speaking communities and first-generation communities. I think it is important to get the support that you need in order to be successful, even if finding the community that is the right fit for you can be challenging.

What does it mean to you, to be queer in STEM?

Being queer in STEM is an opportunity to see that science can be understood from different perspectives. I’m now in a position where I can support more people and help early career researchers. This is my main motivation to do this job right now besides my scientific curiosity. As I mentioned before, I grew up without role models, but now I’m proud to be a queer scientist, and I hope this can serve as a motivation for others to be proud of themselves. A major concern right now in the US, and also in the world, is that since we are in these positions of visibility some people want us to hide. I like to use the phrase “we exist, we resist”. What I mean is that we have to increase our visibility because we have always existed and we will always exist, and I believe that together as a community we will get through these barriers. We must use our privileges and visibility to demand safe spaces and inclusive environments for all.

*This interview was conducted by Associate Editor Laura Rodríguez Pérez*.

